# Isolation of a novel Rhabdovirus from an insectivorous bat (*Pipistrellus kuhlii*) in Italy

**DOI:** 10.1186/s12985-018-0949-z

**Published:** 2018-02-17

**Authors:** Davide Lelli, Alice Prosperi, Ana Moreno, Chiara Chiapponi, Anna Maria Gibellini, Paola De Benedictis, Stefania Leopardi, Enrica Sozzi, Antonio Lavazza

**Affiliations:** 1 Istituto Zooprofilattico Sperimentale della Lombardia e dell’Emilia Romagna (IZSLER), Via Bianchi 9 -, 25124 Brescia, Italy; 2 Wildlife Rehabilitation Center WWF of Valpredina via Pioda n.1, 24060 Cenate Sopra (BG), Bergamo, Italy; 3 Istituto Zooprofilattico Sperimentale delle Venezie (IZSVE), Viale dell’Università, 10 - 35020 Legnaro (PD), Padova, Italy

## Abstract

**Background:**

Rhabdoviridae is one of the most ecologically diverse families of RNA viruses which can infect a wide range of vertebrates and invertebrates. Bats, among mammals, are pointed to harbor a significantly higher proportion of unknown or emerging viruses with zoonotic potential. Herein, we report the isolation of a novel rhabdovirus, detected in the framework of a virological survey on bats implemented in North Italy.

**Methods:**

Virus isolation and identification were performed on samples of 635 bats by using cell cultures, negative staining electron microscopy and PCRs for different viruses. NGS was commonly performed on cell culture supernatants showing cytopathic effect or in case of samples resulted positive by at least one of the PCRs included in the diagnostic protocol.

**Results:**

A rhabdovirus was isolated from different organs of a *Pipistrellus kuhlii*. Virus identification was obtained by electron microscopy and NGS sequencing. The complete genome size was 11,774 nt comprised 5 genes, encoding the canonical rhabdovirus structural proteins, and an additional transcriptional unit (U1) encoding a hypothetical small protein (157aa) (3’-N-P-M-G-U1-L-5′). The genome organization and phylogenetic analysis suggest that the new virus, named Vaprio virus (VAPV), belongs to the recently established genus Ledantevirus (subgroup B) and it is highly divergent to its closest known relative, Le Dantec virus (LDV) (human, 1965 Senegal). A specific RT-PCR amplifying a 350 bp fragment of the ORF 6 gene, encoding for L protein, was developed and used to test retrospectively a subset of 76 bats coming from the same area and period, revealing two more VAPV positive bats.

**Conclusions:**

VAPV is a novel isolate of chiropteran rhabdovirus. Genome organization and phylogenetic analyses demonstrated that VAPV should be considered a novel species within the genus Ledantevirus for which viral ecology and disease associations should be investigated.

## Background

The *Rhabdoviridae* family, within the *Mononegavirales* order, is one of the most ecologically diverse families of viruses with high clinical importance. Rhabdoviruses have a wide geographic distribution and can infect a wide range of plants and animals, including mammals, birds, reptiles and fish. Furthermore, it can infect arthropods, which may act as vectors for the viral transmission [1, 2]. The non-segmented single stranded RNA genome of 11–16 Kb is packaged within the distinct bullet-shaped viral particles (with envelope). This genome comprises the genes encoding for five structural proteins: nucleoprotein (N), phosphoprotein (P), matrix protein (M), glycoprotein (G) and the large protein (L, RNA-dependent RNA polymerase), which is the most highly conserved protein. The genome of rhabdoviruses may also contain additional ORFs encoding putative proteins, which have mostly unknown functions [1, 2].

The family *Rhabdoviridae* accounts for more than 130 viral species recognized by the International Committee in Taxonomy of Viruses (ICTV). These are divided into 18 genera based on their phylogeny, genome’s organization, host and/or vectors (Almendravirus, Curiovirus, Cytorhabdovirus, Dichoravirus, Ephemerovirus, Hapavirus, Ledantevirus, Lyssavirus, Novirhabdovirus, Nucleorhabdovirus, Perhabdovirus, Sigmavirus Sprivivirus, Sripuvirus, Tibroviurus, Tupaiavirus, Varicosavirus and Vescivulovirus) [3].

Bats are well-known natural hosts for emerging viruses with zoonotic potential, due to their abundance, geographical distribution and zoo-ecological characteristics [4]. A recent comprehensive analysis of mammalian host–virus relationships demonstrated that bats harbor a significantly higher proportion of zoonotic viruses and are more likely to carry unknown pathogens compared to all other mammalian orders [5].

Rhabdoviruses are frequently found in bats worldwide, which mainly belong to the genus of *Lyssavirus* detected in bats worldwide [6]. In Europe, rabies-related lyssaviruses are currently the only proven zoonotic pathogens founds in bats, with more than 900 cases reported since infected bats were first found in Europe in 1954 [7]. Other than lyssaviruses, there are only few reported cases of rhabdoviruses in bats in Europe [8–10]. All these findings consist of short genomic sequences with no isolated strains, which are therefore unsuitable for solid phylogenetic hypothesis as well as for further characterization and clarification of its host range, pathogenicity and zoonotic potential.

In this study, we report the isolation and the full-genome characterization of a novel rhabdovirus, detected from an insectivorous bat *(Pipistrellus kuhlii)* in Northern Italy, which was tentatively named Vaprio virus (VAPV) according to the town where the infected bat was found. Genome organization and phylogenetic analyses suggest that VAPV belongs to the subgroup B of the recently established genus *Ledantevirus*. VAPV appears to be only distantly related to its closest known relative, Le Dantec virus (LDV) (human, 1965 Senegal), an enigmatic virus associated with human disease for which the clinical and epidemiological aspects are still uncertain [11, 12].

## Methods

### Sampling

From January 2010 to May 2017 a total of 635 bats have been sampled in the context of surveillance implemented in Lombardy and Emilia Romagna region (Northern Italy) with regards to emerging viruses of bats, which was launched on 2009 [13, 14]. Fresh fecal samples and dead bats of different species were collected for virological investigations from wild animal rescue/rehabilitation centers and known bat colonies. The bats were taxonomically identified based on their morphologic characteristics, according to the European bat identification keys [15]. The carcasses have been necropsied and tissue samples were collected for further examinations, which was particularly focused on virus detection.

### Virological analysis

After necropsy, organ samples (lungs, hearth, kidney, brain and intestines) and the oropharyngeal swab were homogenized in minimal essential medium (1 g/10 mL), which contained antibiotics and were centrifuged at 3000 g for 15 min.

All samples were inoculated in confluent monolayers of VERO (African green monkey) cells, incubated at 37 °C with 5% CO_2_ and observed daily for 7 days for cytopathic effects (CPEs). In the absence of CPEs, the cryolysates were sub-cultured twice onto fresh monolayers. The supernatant from cell cultures showing CPEs were submitted for viral identification with negative-staining electron microscopy (nsEM) by using the Airfuge method [16].

### Molecular analysis

Viral RNA was extracted from 200 μl of positive cell culture supernatants by Trizol. Sequencing libraries were made with TruSeq RNA Library Preparation Kit v 2 (Illumina Inc. San Diego, CA, USA) according to the manufacturer’s instructions. Libraries were sequenced on a MiSeq Instrument (Illumina Inc. San Diego, CA, USA) by using a Miseq Reagent Kit v2 in a 250-cycle paired-end run. Data were assembled de novo by the NextGen DNASTAR (DNASTAR, Madison, WI, USA) application and were analyzed by the Lasergene Package software (v12.0). Complete genome sequences of rhabdoviruses were obtained from the GenBank database. The multiple sequence alignment was performed using ClustalW software implemented in BioEdit version 7.0.9.0. The maximum likelihood phylogenetic tree was performed using IQtree software [17] and the model finder was used to determine the best model according to BIC [18]. The neighbor-joining phylogenetic tree was performed using MEGA 6 software to compare topologies [19].

Successively, an end-point one-step RT-PCR was developed using the complete viral genome sequenced. Primers IZSLER-VAPV F (5′- TTG TTC CTC TGT TCA GCG GTC -3′) and IZSLER-VV R (5′- TCC GCC TAA TTG TCC ATT CC -3′) were designed on a conserved 350 bp region of the ORF-6 gene, which encodes for the RNA-dependent RNA polymerase (L protein) of the VAPV. Total RNA was extracted from the tissue samples and the cell culture supernatants using Qiazol®, following the manufacturer’s instructions. The PCR assay was performed with the QIAGEN® OneStep RT-PCR master mix (Qiagen, Hilden, Germany). The reaction mixture contained 0.6 μM of sense and antisense primer and 5 μl of the RNA template in a final volume of 25 μl. PCR conditions were optimized using the bats’ organs and cell culture samples, which were known to be positive and negative for VAPV infections. The thermal cycling conditions consisted of 30 min at 50 °C for reverse transcription, 15 min at 95 °C for the initial enzyme activation and 7 touchdown thermal cycles (94 °C for 30 s; from 63 °C to 54 °C for 45 s; and 72 °C for 45 s). This was followed by 35 thermal cycles (94 °C for 30 s; 54 °C for 45 s; and 72 °C for 45 s), with a final elongation step of 72 °C for 5 min. Amplicons were visualized on 2% agarose electrophoretic gels, which were stained with EuroSafe Fluorescent Nucleic Acid Stain (Euroclone, Milan, Italy) used at a 1× concentration.

The RT-PCR here developed was subsequently used to further asses the circulation of VAPV especially among bats originating from the same area and almost same period, with particular attention for individual possibly exposed to the virus as admitted to the same rescue center of the control case. A subset of samples from 76 bats (lungs, hearth, kidney, brain and intestines) were preliminary selected and screened, including 32 *Pipistrellus kuhlii*, 2 *Plecotus auritus*, 30 *Hypsugo savii* and 12 non-identified bats of the genus *Pipistrellus*.

## Results

### Virus isolation and identification

The case concerns an adult female of *Pipistrellus kuhlii*, who was found to be spontaneously dead in a wildlife recovery center in Valpredina, Cenate Sopra (BG), Northern Italy. The sick bat was originally found alive on September 29th, 2016 in Vaprio d’Adda village (Bergamo province, northern Italy) by a private citizen who brought it to the center. Anamnesis reported sensory depression and lack of appetite but normal body mass. The death occurred three days after the admission to the rehabilitation center in October 2016. During the necropsy, pathological lesions indicative of infectious diseases were not observed. However, the animal had traumatic lesions on the wing membrane, which were possibly related to a cat bite. Samples collected for virological investigations included a pool of viscera (lung and hearth), the intestinal tract, the kidneys and the brain. Additionally, an oropharyngeal swab from the carcass was taken.

**Fig. 1:**
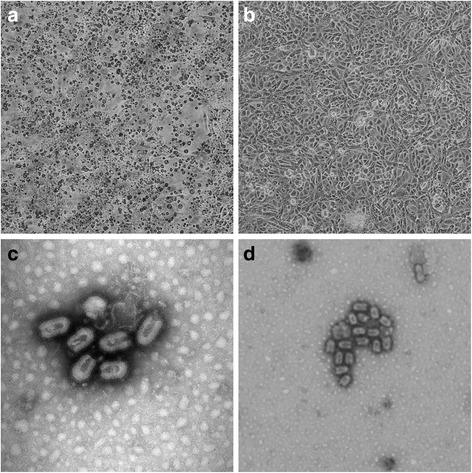
(**a**) CPE with rounded cells fluctuating in the culture medium of Vero cells infected with the pool of bat’s organs (heart and lungs) at 3 days after inoculation (original magnification × 100) (**b**) Mock cells (original magnification × 100). (**c**) and (**d**) Negative-staining electron microscopy showing the presence of virions morphologically related to the Rhabdoviridae family from VERO cell culture. The scale bar in panel A indicates 200 nm, which is 100 nm in panel B

A virus was isolated on VERO cells from the organs’ pool composed by the heart and lungs. The CPE occurred at the third day post-inoculation during the 2° passage, which was characterized by a the diffused degeneration of a monolayer with rounded cells fluctuating in the culture medium (Fig. 1A and B). Furthermore, nsEM performed on cell culture supernatant revealed the presence of many distinct bullet-shaped viral particles, which are related to the Rhabdoviridae family (Fig. 1C and D). Tests aimed to exclude positivity to rabies and there was no relation to the lyssavirus.

### Genome characterization

After NGS sequencing the complete viral genome was obtained from a contig of 11,774 nucleotides, assembly results are shown in Table 1. The virus identification was confirmed and the full genome sequence of the viral strain was determined and compared with those of rhabdoviruses available on GenBank.

**Table 1:** Assembly report of NGS sequencing by NextGen DNASTAR Lasergene Package software (v12.0)

Contig length	Contig length without gaps	Total seq. Length	Number Seq.	Average Coverage
12,120	11,774	26,894,188	177,679	2218.99

**Fig. 2:**
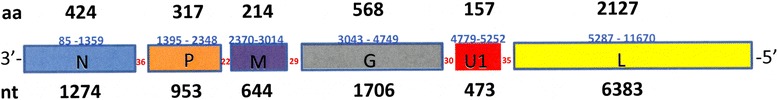
Genome organization of Vaprio virus (VAPV). Letters indicate the canonical rhabdovirus nucleoprotein (N), phosphoprotein (P), matrix (M), glycoprotein (G) and polymerase (L) genes and additional transcriptional unit (U1)

The whole genome comprised of 5 genes encoding the canonical rhabdovirus structural proteins (N, P, M, G and L), with an additional transcriptional unit (U1) encoding a hypothetical small protein of 157 amino acids located between the G and L genes (Fig. 2). The new virus was tentatively named Vaprio virus (VAPV) based on the sampling place of the positive bat, the village of Vaprio d’Adda, Bergamo provincein Northern Italy.

The results of the BLAST analysis showed the highest amino acid sequence identity for each protein of the new VAPV was to two viruses detected in the 1960s, which are named Le Dantec virus (LDV) (human, 1965 Senegal) and Keuraliba virus (KEUV) (gerbil, 1968 Senegal). These were respectively detected in humans and in rodents [12]. The amino acid identity range was from 37% (for the phosphoprotein) to 70% (for the L protein, which is the most highly conserved) (Table 2). If we consider the complete viral genome sequence, BLAST analysis revealed the highest nucleotide identity (65%) to LDV, the prototype strain of the recently established genus of Ledantevirus. The complete genome sequence for VAPV was submitted to GenBank under accession number MG02144.

**Table 2:** Highest amino acid sequence identities for each protein of the novel VAPV

Protein	% similarity (query cover %)	Rhabdovirus strain	GenBank Acession No.
N	64 (100)	Le Dantec virus (LDV)	YP_009361868
	63 (100)	Keuraliba virus (KEUV)	YP_009362195
P	37 (99)	Keuraliba virus (KEUV)	YP_009362196
	34 (99)	Le Dantec virus (LDV)	YP_009361869
M	56 (98)	Le Dantec virus (LDV)	YP_009361870
	53 (98)	Keuraliba virus (KEUV)	YP_009362197
G	64 (86)	Le Dantec virus (LDV)	YP_009361871
	61 (91)	Keuraliba virus (KEUV)	YP_009362198
U1	No significant alignment		NA
L	70 (99)	Keuraliba virus (KEUV)	AJR_28566
	69 (99)	Le Dantec virus (LDV)	AJR_28452

Note: N = nucleoprotein; P = phosphoprotein; M = matrix protein; G = glycoprotein; U1 = additional transcriptional unit; L = large protein (RNA-dependent RNA polymerase) and NA = Not applicable

**Fig. 3:**
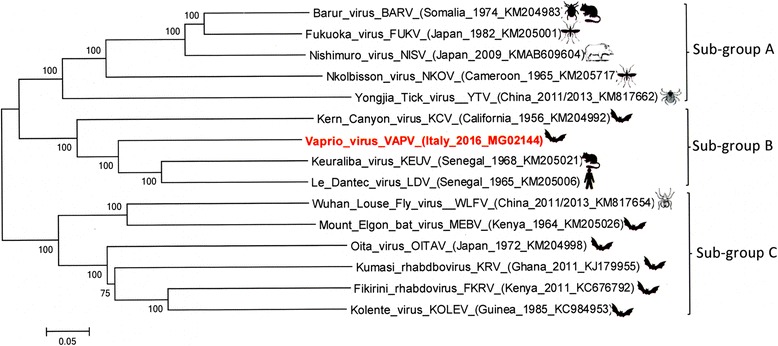
Phylogenetic tree based on the complete genome (11,774 nt) of the new *Vaprio virus* (VAPV) performed with all the 14 known viral species belonging to the genus Ledantevirus (family Rhaboviridae) Legend fig. 3: The tree was performed using the maximum likelihood method, which is namely the GTR-G model within the IQ-tree software with a bootstrap of 1000 replicates. The VAPV strain is reported in red.

Phylogenetic analysis performed with all the 14 known viruses belonging to the Ledantevirus genus (Fig. 3) suggested that VAPV is a new member of the subgroup B of this recently established genus. Moreover, VAPV satisfies the species demarcation criteria of the international Committee on the Taxonomy of Viruses (ICTV), *Rhabdoviridae* Study Group for classification in the genus of Ledantevirus [20].

### VAPV tissue tropism and preliminary VAPV screening

In order to obtain more information on the tissue target, the VAPV end-point one-step RT-PCR developed during the study was used to further screen cultured cells and the original tissue samples from the control bat. The RT-PCR specificity was verify and confirmed by submitting the generated amplicons to the genome sequencing. A strong PCR positivity was obtained from the pool of organs (hearth and lungs), the kidneys and the brain. In comparison, a weak positivity was achieved from the intestine. The oropharyngeal swab sample performed on the defrosted bat was also positive.

In order to obtain preliminary information on the epidemiology of this new virus and particularly to screen its circulation, the newly developed RT-PCR was used to investigate 76 bats (organs samples) collected in the same period and the same are in Northern Italy. Two more VAPV-infected bats, one (*Hypsugo savii*) from the same rescue center and one (*Pipistrellus kuhlii)* from another one*,* were identify by PCR and then confirmed by the genome sequencing of the generated amplicons (350 bp).

## Discussion

The exceptional ecological value of both insectivorous and fruit bats has been finally and rightly accepted by everyone. In the meanwhile, the interest for bat-associated viruses has increased significantly during the last decade, leading to a growing number of novel viruses being discovered in several bat species. In this study, we described the isolation and the complete genome characterization of a novel rhabdovirus, which is tentatively named Vaprio virus (VAPV), detected from a Kuhl’s pipistrelle in Northern Italy.

The obtaining of the complete genomic sequence of VAPV clearly demonstrated that this new virus is genetically related to the LDV human strain found in Senegal in 1965. However, our strain showed large nucleotide and amino acid divergence from LDV and other strains belonging to the genus of Ledantevirus, of which LDV is the prototype. Phylogenetically, VAPV clusters within the subgroup B of the genus Ledantevirus are comprised of the LDV, KEUV and Kern Canyon viruses (KCV) (California, 1956) [20]. Moreover, VAPV showed the same genome organization of LDV, KEUV and KCV, which are the only three viruses out of the 14 members of the Ledantevirus genus to contain an additional gene (Fig. 2). This additional gene encodes for a protein of unknown function, with no significant similarity to any other previously identified protein sequence [21].

VAPV widely fulfills four of the five species demarcation criteria of the ICTV Rhabdoviridae Study Group for Viruses classification [21]. Therefore, it could be considered a different species within the genus Ledantevirus based on: A) minimum amino acid sequence divergence of 7% in L proteins (VAPV vs KEUV = 30%); B) minimum amino acid sequence divergence of 15% in G proteins (VAPV vs LDV = 46%); C) same genome organization as other ledanteviruses (Fig. 2); D) occupancy in a different ecological niche than other ledanteviruses, as evidenced by detection in a new host (*Pipistrellus kuhlii*) and area (Italy). Only the last criterium i.e. possibility to differentiate by serological tests was not evaluated due to the lack of availability of the other species of genus ledantevirus. However, the high divergence of the VAPV G sequence, the only surface protein, strongly suggests that VAPV should also be serologically distinct.

Within the Rhabdoviridae family, lyssaviruses pose the greatest concern for public health and have been frequently associated with bat hosts worldwide. Besides lyssaviruses, the known rhabdoviruses which have been found in bats are Oita virus (OITAV) (Japan, 1972) [22], Mount Elgon bat virus (MEBV) (Kenya, 1964) [23], Kern Canyon virus (KCV) (California, 1956) [24], Kolente virus (KOLEV) (Guinea, 1985) [25], Fikirini rhabdovirus (FKRV) (Kenya, 2011) [26] and Kumasi rhabdbovirus (KRV) (Ghana, 2011) [27]. All these isolated viruses belong to the subgroup A of the genus Ledantevirus with the exception of KCV, which belongs to the subgroup B. None of them have been implicated as a cause of human or animal disease. However, OITAV and MEBV have shown the ability to cause neurological disease after intra-cranial inoculation in mice [22, 28]**.** Recently, a new putative member of the rhabdoviral genus of Ledantevirus has been identified in Uganda, which is named Kanyawara virus (KIAV). This is genetically related to MEBV and infects Nycteribiid bat flies [29].

In Europe, sequences of rhabdovirus have been detected in oropharyngeal swabs from German, Danish and Spanish bats [8, 10]. These viral sequences show the highest homology with rhabdoviruses from Diptera, which suggests that they might be also associated with arthropods. However, these findings consist only of short genomic sequences and none of them has been ever isolated. Furthermore, VAPV was isolated from the organ of bats and showed the highest nucleotide similarity to LDV, which has been isolated from the serum of a 10-year-old female with fever and hepatosplenomegaly, supporting its pathogenicity for humans [12].

Despite the fact that the new virus was isolated only from a pool of bat viscera including heart and lungs, VAPV-RNA was also detected by RT-PCR in other organs and in an oropharyngeal swab. However, additional studies are needed to establish whether the presence of VAPV RNA in the oro-pharyngeal cavity or in bat secretions is associated with the excretion of mature infectious virions.

Only one bat out of several hundred samples (> 600 bats) was tested positive by cell culture isolation for this novel virus. One of the possible reason of a such low rate of detection and cell culture isolation could be the lack of vitality of Rhabdoviruses, which are well-known to poorly resist in not very fresh and well preserved samples. However, the preliminary screening by the VAPV-specific RT-PCR of a first subset of 76 bats taken in the same area in Northern Italy and almost same period yielded the identification of two more VAPV-infected bats (*Pipistrellus kuhlii* and *Hypsugo savii*).

Even if the geographical and host range of VAPV is still to be defined, the identification of *P. kuhlii* as a potential reservoir for new viral agents, such as VAPV, could represent a significant eco-epidemiological information since this is the most common bat species in the Italian urban areas. Moreover, in the last decades, *P. kuhlii* has showed an extraordinary range expansion due to its ecological flexibility and capability to successfully exploit human settlements, roosting in buildings and foraging in streetlamps [30]. Therefore, the knowledge of the high biodiversity of bats, the broad geographical distribution and the genetic diversity of bat-associated viruses is crucial for a comprehensive study from which viral discovery studies, viral disease prevention and biological conservation issues can benefit.

## Conclusions

A novel rhabdovirus, tentatively named Vaprio virus (VAPV), has been identified from *Pipistrellus kuhlii* in Italy. Other than lyssaviruses, this strain represents the only bat-borne rhabdovirus fully sequenced and isolated in cell cultures in Europe. Genome organization and phylogenetic analyses demonstrated that VAPV should be considered a novel species within the genus of Ledantevirus. In addition, a VAPV specific RT-PCR has been developed, which is available for further diagnostic and epidemiological investigations.

## Abbreviations

BG: Bergamo
BLAST: basic local alignment search tool
CPE: cytopathic effect
FKRV: Fikirini rhabdovirus
G: glycoprotein
ICTV: the International Committee in Taxonomy of Viruses
IZSLER: Istituto Zooprofilattico Sperimentale della Lombardia e dell’Emilia Romagna
KCV: Kern Canyon virus
KEUV: Keuraliba virus
KOLEV: Kolente virus
KRV: Kumasi rhabdbovirus
L: large protein
LDV: Le Dantec virus
M: matrix protein
MEBV: Mount Elgon bat virus
N: nucleoprotein
NGS: Next Generation Sequencing
nsTEM: negative staining Transmission Electron microscopy
OITAV: Oita virus
ORF: open reading frame
P: phosphoprotein
RT-PCR: reverse transcriptase-polymerase chain reaction
U1: transcriptional unit
VAPV: Vaprio virus
